# IQGAP3 Promotes EGFR-ERK Signaling and the Growth and Metastasis of Lung Cancer Cells

**DOI:** 10.1371/journal.pone.0097578

**Published:** 2014-05-21

**Authors:** Ying Yang, Wei Zhao, Qing-Wen Xu, Xiao-Song Wang, Yu Zhang, Jun Zhang

**Affiliations:** Department of Immunology, School of Basic Medical Sciences, Key Laboratory of Medical Immunology of Ministry of Public Health, Peking University Health Science Center, Beijing, P. R. China; Cincinnati Children’s Hospital Medical Center, United States of America

## Abstract

Proteins of the IQGAP family display complicated and often contradictory activities in tumorigenesis. IQGAP1 has well documented oncogenic potential and IQGAP2 has putative tumor-suppressive function. IQGAP3 is the latest addition to this family and its role in cancer development remains to be defined. Here we demonstrate IQGAP3 expression is markedly increased in lung cancer tissues at both mRNA and protein levels. Overexpression of IQGAP3 promoted tumor cell growth, and migration and invasion, whereas knockdown of IQGAP3 exhibited opposite effects. Moreover, suppression of IQGAP3 in a lung cancer cell line caused a reduction in the tumorigenicity of these cells in lung tissue after intravenous injection. Furthermore, we showed that IQGAP3 is able to interact with ERK1 and enhance its phosphorylation following treatment with EGF. These data suggest that IQGAP3 may contribute to the pathogenesis of lung cancer by modulating EGFR-ERK signaling.

## Introduction

Lung cancer ranks first in cancer related mortality both in China and worldwide [1], [2]. In China, there are approximately 300,000 new lung cancer cases and more than 250,000 deaths from this disease each year [3]. Histologically, as many as 85% of lung cancers are non-small cell type lung cancer (NSCLC), and the majority of these are either adenocarcinoma or squamous cell carcinoma [4]–[7]. As lung cancer may be occult, most patients are inoperable and have metastases to regional lymph nodes or to distant sites at the time of diagnosed. The NSCLC patients with distant metastases survive for a short time (from 9 to 12 months) [5], [8]. There is therefore an urgent need to unravel the molecular mechanisms which lead to invasion and metastasis in lung cancer [9], [10]. Such information will facilitate the development of novel therapies allowing improvement of the outcome in lung cancer patients [11], [12].

Therapeutic approaches against EGF or EGFR represent a promising direction for lung cancer therapy [13], [14]. EGFR is expressed in normal cells of epidermal, mesenchymal and neurogenic origin, and its activation is strictly controlled in normal tissues [15], [16]. However, binding of EGFR by its ligand results in receptor homo- or heterodimerization and activation of its intrinsic tyrosine kinase activity [17]. The downstream signaling cascade is thus initiated, ultimately leading to changes in such cell behaviors as proliferation, migration and differentiation [15], [16]. Importantly, constitutive activation of EGFR or enhanced EGF signaling is frequently found in different types of cancers, especially in lung cancer, where it is associated with cancer initiation, tumor growth/progression, metastasis and poor prognosis [17]–[20].

The IQGAP family of proteins is well conserved in organisms from yeast to mammals [21]. It comprises three members, IQGAP1, IQGAP2 and IQGAP3 [22]–[24]. Among these, IQGAP1 is the best studied [25]. The name IQGAP is derived from the multiple functional domains these molecules harbor such as four IQ motifs and a RasGAP-related domain (GRD) [26], [27]. IQGAP1 also contains putative coil-coil homodimerization domains, a tryptophan repeat motif (WW) of unknown function, a calponin-homology domain (CHD) that interacts with F-actin, and a RasGAP_C-terminus (RGCt) that interacts with numerous proteins including E-cadherin and β-catenin [27]. IQGAP1 has been suggested to function in regulation of the cytoskeleton and cell migration [28]–[30]. There is also evidence which indicates IQGAP1 plays a role in cancer progression [31], [32]. In contrast, IQGAP2 seems to act as a tumor suppressor [33]. IQGAP3 is the latest addition to this family [24]. Data currently available suggest that it is involved in the proliferation of epithelial cells [34], [35], however its role in tumorigenesis remains to be determined. In the current study, we provide the first evidence that IQGAP3 promotes lung cancer growth and metastasis by enhancing EGFR-mediated ERK signaling. IQGAP3 may therefore play a role similar to that of IQGAP1 in tumorigenesis.

## Materials and Methods

### Ethics Statement

This study was approved by the Ethics Committees of Peking University Health Science Center (Beijing, China) and the 306th Hospital of the People’s Liberation Army of China (Beijing, China). For animal studies, all efforts were made to minimize suffering and when observed suffering was too great, humane euthanasia was used. Written consent was obtained from individual patients for use of tissue specimens.

### Cell Lines and Patient Specimens

A549 and Hela cells were cultured in RPMI 1640 medium supplemented with 10% fetal bovine serum (Life Technologies, Carlsbad, CA, USA). HEK293T cells were cultured in DMEM medium supplemented with 10% fetal bovine serum. Cells were cultured at 37°C in a humidified 5% CO_2_ atmosphere.

For cell signaling assays, cells were serum deprived (0.5% fetal bovine serum) for 16 h prior to stimulation with 100 ng/ml EGF (Peprotech, Rockville, NJ, USA) for different lengths of time as indicated.

25 paired lung tumor tissue and adjacent normal tissue specimens were obtained from the 306th Hospital of the People’s Liberation Army of China.

### Plasmids and Transfection

Myc-pCAGGS-IQGAP3 and control pCAGGS vectors were kindly provided by Dr. Kozo Kaibuchi. HA-tagged ERK1 or ERK2 expressing vectors were constructed by standard molecular techniques and verified by sequencing.

Cells were transfected with jetPRIME transfection reagent (Polyplus Transfection, Illkirch, France).

### siRNA Transfection and Lentiviral Infection

Small interfering RNA oligonucleotides (oligos) against IQGAP3 or control siRNA oligonucleotides were obtained from GenePharma Co., Ltd (Shanghai, China). The targeting sequences of these siRNAs were as follows: si IQGAP3-1∶5′- GAGCCAACCAGGACACUAA-3′; si IQGAP3-2∶5′- GGCAGAAACUAGAAGCAUA-3′; control siRNA:5′- UUCUCCGAACGUGUCACGU-3′. siRNA oligos(50 nM)were transfected into A549 cells with jetPRIME transfection reagent.

Alternatively, the siRNA target sequence was cloned into the lentiviral vector pLL3.7. Upon sequence verification, the shRNA plasmid and packaging vectors psPAX2 and pLP/VSVG were transfected into the packaging cell line HEK293T using jetPRIME. The medium was changed 8 h post-transfection. 48 hours later, viral supernatant was harvested and incubated with the A549 cell line in the presence of 8 µg/ml polybrene (Sigma-Aldrich, Saint Louis, MO, USA). For stably transfected cell line selection, GFP fluorescence was used as a sorting marker. Cells with >75% infection efficiency were used for further analysis.

### Reverse Transcriptase-PCR and Real-time PCR

TRIzol (Life Technologies) was used to isolate total RNA and then reverse transcribed into cDNA by the Reverse Transcription System (Promega, Madison, WI, USA). Quantitative realtime PCR was carried out on a Bio-Rad Real-Time PCR system. The sequences of the realtime primers for IQGAP3 were as follows: forward, 5′-GTTCATCCATAGAGCCTGCCA-3′; reverse, 5′-GCGATGCTCTCACCAATAAGG-3′. The realtime primers for GAPDH have been described before [36]. Gene expression was quantified as the yield of IQGAP3 relative to that of GAPDH.

### Immunoprecipitation and Western Blot Analysis

Myc-IQGAP3 and HA-ERK1 or HA-ERK2 constructs were transfected into HEK293T cells. Cells were harvested and lysed in lysis buffer with proteinase inhibitor cocktail (Roche, Basel, Switzerland) and phenylmethylsulfonyl fluoride. Then the cell lysates were incubated with mouse anti-HA mAb (Sigma-Aldrich), anti-Myc mAb (Sigma-Aldrich) or a control antibody (mIgG) (Sigma-Aldrich) and protein-A Sepharose (GE Healthcare, USA) and resolved by SDS-PAGE. For endogenous immunoprecipitation, cell lysate was prepared from A549 cells and immunoprecipitated with rabbit anti-ERK1 mAb (Santa Cruz Biotechnology, Santa Cruz, CA, USA).

For western blot analysis, equal amounts of protein from each sample was loaded and resolved with SDS-PAGE and transferred to blots. After blocking, blots were probed with indicated antibodies and evaluated with the Odyssey Imaging System (LICOR Bioscience, Lincoln, NE, USA). Antibodies used included anti-GAPDH (Bioworld Technology, Inc., St Louis Park, MN, USA), anti-IQGAP3 (Sigma-Aldrich), anti-ERK (Cell Signaling, Beverly, MA, USA), anti-phospho-Erk^Thr202/Tyr204^ (Cell Signaling), anti-phospho-p38^Thr180/Tyr182^ (Cell Signaling), anti-phospho-Akt^Ser473^ (Cell Signaling), anti-Myc, and anti-HA.

### Tissue Microarray and Immunohistochemistry

A lung cancer tissue microarray (TMA) was purchased from Chaoying Biotechnology Co. (Xi’an, China). Sections were incubated with anti-IQGAP3 antibody (1∶50 dilution) overnight at 4°C. The primary antibody was identified using a horseradish peroxidase enzyme-labeled polymer conjugated to goat anti-mouse secondary antibody (Promega). The stained sections were reviewed and scored by a pathologist. The negative control was normal human lung tissue.

### Cell Proliferation Assay

Cells were plated in 96-well plates at a density of 3×10^3^ cells/well. Cell proliferation was evaluated every 24 h using the Cell Counting Kit-8 (CCK-8) (Dojindo Laboratories, Japan). Each experiment was performed in triplicate.

### Cell Migration and Invasion Assay

Migration assays were performed in a 24-well Chemotaxis chamber (8-µm pore size, Corning Life Sciences, Corning, NY, USA) coated with 10 µg/ml fibronectin (Sigma–Aldrich). Cells (3∼5 ×10^4^ cells/well) in 200 µl serum-free RPMI 1640 were seeded into the upper chamber. Then 600 µl RPMI 1640 with 10% fetal bovine serum or 100 ng/ml human EGF were added into the lower chamber. Cells in the upper chamber were removed using a cotton swab after 20 h of incubation at 37°C and 5% CO_2_. Cells attached to the bottom of the membranes were fixed with methanol and stained by crystal violet. The degree of migration was expressed as the average number of cells in six 10× fields.

Cell invasion assays were carried out in essentially the same manner as the migration assay, except that the upper chamber was covered with 30 µl Matrigel (0.5 mg/ml; BD Biosciences). Experiments were performed in triplicate.

### Luciferase Reporter Assay

The Elk-1 transcriptional activity were measured with the Dual-Luciferase Reporter Assay System (Promega). IQGAP3 siRNA or NC siRNA was co-transfected into A549 cells with the plasmids pFR-Luc (reporter plasmid), pFA2-Elk-1 (transactivator plasmid) or pRL-TK. Twenty-four hours later, cells were serum-starved for 16 h, then stimulated with or without 100 ng/ml EGF for 6 h. Cells were harvested and lysed. All reporter assays were repeated at least three times in triplicate. Firefly luciferase and Renilla luciferase activity were measured with a Centro LB960 luminometer (Berthold Technologies, Bad Wildbad, Germany). Firefly luciferase activity was calculated and normalized based on Renilla luciferase activity. Luciferase activity in control siRNA-transfected cells without EGF stimulation was set as 1.

### Tumor Metastasis Analysis *in vivo*

NOD/SCID mice were purchased from Vital River Laboratory Animal Technology Co. Ltd (Beijing, China). Mice were raised in a pathogen-free facility at Peking University Health Science Center (Beijing, China).

A549 cells were infected with control shRNA or shIQGAP3 lentiviruses. Cells (1.5×10^6^ ) in 0.1 ml PBS were injected into the tail vein of 6-week-old NOD/SCID mice. Six weeks after infection, mice were sacrificed by cervical dislocation. Then lungs were removed, weighed, photographed, and fixed in 4% formalin. Tissue sections were also stained with hematoxylin-eosin (H&E) for histologic evaluation.

### Statistical Analysis

Statistical analysis was carried out using SPSS, version 13.0 (SPSS, Inc.). The Student’s t-test was used to compare cell proliferation, migration, and invasion between two independent groups. The Chi-square test was performed to determine differences in patient’s age, gender, tumor stage and histology among groups with higher, equal or lower IQGAP3 expression in tumor versus adjacent non-cancerous tissues. *P* values <0.05 were considered statistically significant.

## Results

### IQGAP3 Expression is Upregulated in Lung Cancer Tissue

**Figure 1:**
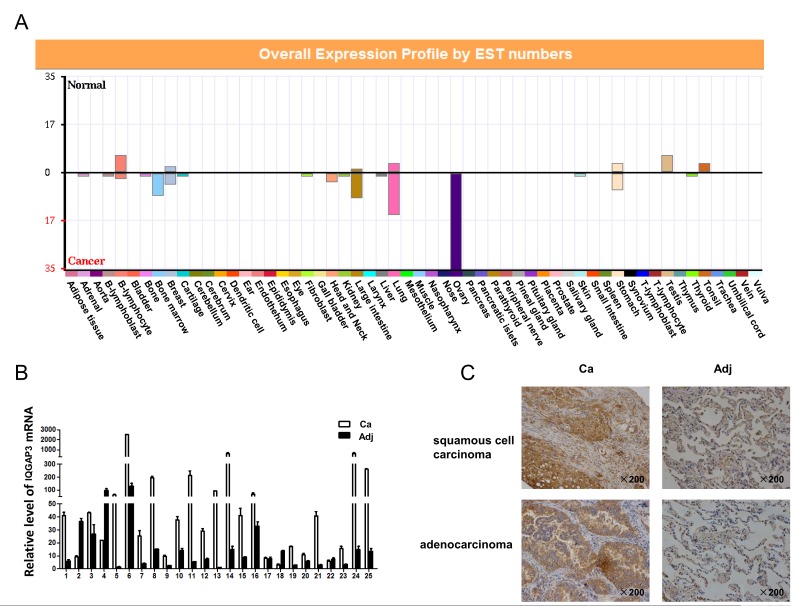
Increased expression of IQGAP3 in lung cancer tissues. (A) EST expression analysis of the IQGAP3 gene in normal and cancerous tissues based on the ECgene database. (B) Quantitative real-time PCR for IQGAP3 expression in 25 pairs of lung cancer versus adjacent non-cancerous tissues. IQGAP3 expression was normalized against GAPDH. Relative levels were calculated for each sample, and a value of 1 was assigned for adjacent non-cancerous tissue of patient 13. Experiments were repeated in triplicate. Data from one representative experiment are presented as mean±SD. (C) Immunohistochemical analysis of IQGAP3 protein expression in lung cancer versus adjacent non-cancerous tissues. Representative images are shown for a squamous cell carcinoma and an adenocarcinoma. Ca, Cancer tissue; Adj, adjacent non-cancerous tissues. Magnification, ×200.

A potential role in tumorigenesis was first suggested for IQGAP3 by bioinformatic analyses of IQGAP3 expression in tumor tissue. ECgene (http://genome.ewha.ac.kr/ECgene/) showed elevated levels of IQGAP3 in a variety of tumor tissues or cells of origin in the ovary, lung, large intestine, stomach, bone marrow and breast (Figure 1A). RT-PCR was subsequently performed to verify these data. While IQGAP3 expression in normal tissues was restricted to the colon, small intestine and testis, high levels of IQGAP3 mRNA were observed in a number of tumors including lung cancer, hepatocellular carcinoma, renal cancer, gastric cancer, bladder cancer, colon cancer and leukemia (data not shown). Moreover, we saw production of autoantibodies against IQGAP3 in a significant fraction of patients with IQGAP3-positive lung cancer [37]. This prompted us to further inquire into the activity of this molecule in lung cancer development. IQGAP3 was upregulated at mRNA level in 20 lung cancer tissues as compared to adjacent non-cancerous tissues in 25 paired samples by real-time PCR analysis (Figure 1B). We next examined IQGAP3 protein expression in a tissue array containing 89 paired lung cancer tissues. Elevated levels of IQGAP3 protein were observed in 80 cancer tissues (Figure 1C and Table S1). Of note, statistical analysis showed that there was no significant correlation between IQGAP3 protein upregulation and parameters such as age, gender or histological grade (Table S1).

### Alteration of IQGAP3 Expression Modulates the Growth, Migration and Invasion of Tumor Cells

**Figure 2:**
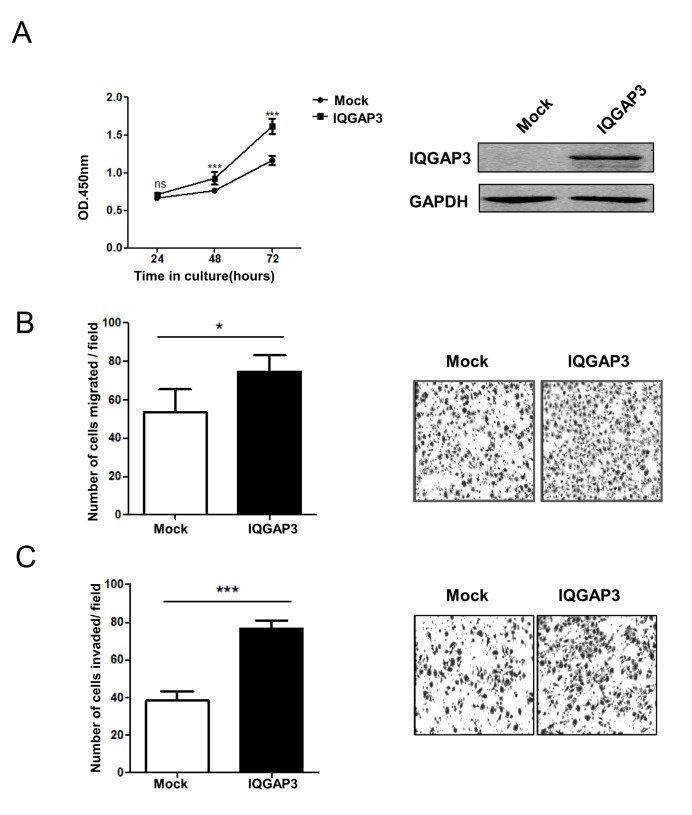
Enhanced cell growth, migration and invasion with enforced expression of IQGAP3. Myc-IQGAP3 or control vectors were transfected into Hela cells and then cells were harvested at 24 h after transfection for migration and invasion assay. Cells for proliferation assay was harvested at indicated time point. (A) IQGAP3 protein expression of transfectants was verified by Western blotting. Cell proliferation was assayed using the Cell Counting Kit-8. (B, C) Cell migration and invasion assays were performed using Chemotaxis chambers with or without coating with Matrigel. Each assay was repeated at least 3 times. Data from one representative experiment are presented as mean±SD. *, *P*<0.05; ***, *P*<0.001.

To investigate the functional consequence of upregulated IQGAP3 expression in cancer, we monitored changes in cell behavior following alteration of its expression level in cancer cell lines. IQGAP3 was first overexpressed in Hela cells, which had a relatively low level of endogenous IQGAP3 expression. Overexpression of IQGAP3 promoted cell proliferation in these cells (Figure 2A). In addition, enforced expression of IQGAP3 resulted in enhanced cell migration and invasion (Figure 2B, C).

**Figure 3:**
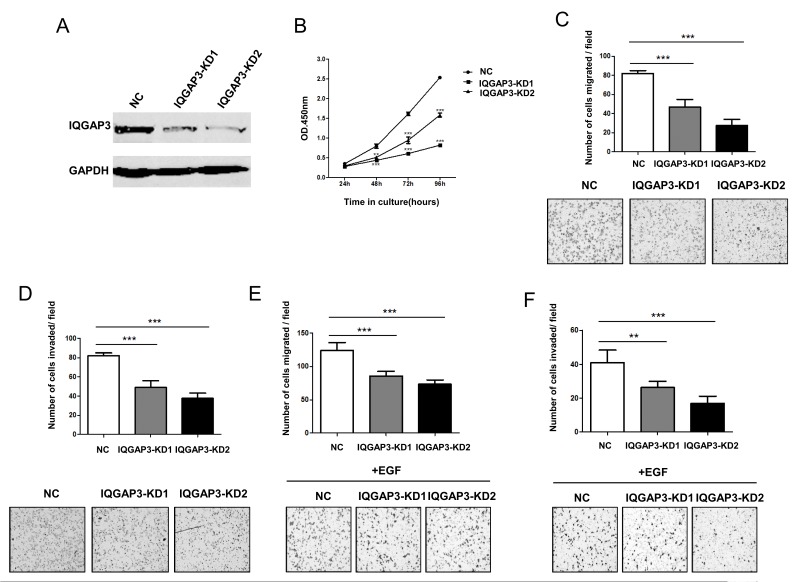
Reduced cell growth, migration and invasion upon inhibition of IQGAP3 expression. A549 cells were transfected with shRNA constructs to knockdown endogenous IQGAP3 expression. (A) IQGAP3 protein levels following infection with control (NC) or two different shIQGAP3 lentiviruses (KD1 and KD2). (B) Proliferation was assayed using the Cell Counting Kit-8 for lentivirus-infected A549 cells. (C, E) Migration of lentivirus-infected A549 cells in the chemotaxis chamber assay in the absence (C) or presence (E) of human EGF (100 ng/ml). (D, F) Invasion of lentivirus-infected A549 cells across Matrigel in the absence (D) or presence (F) of human EGF. Each assay was repeated at least 3 times. Data from one representative experiment are presented as mean±SD. *, *P*<0.05;**, *P*<0.01;***, *P*<0.001.

To further evaluate the effects of IQGAP3 on cancer cell growth and migration, IQGAP3 expression was knocked down in the lung cancer cell line A549, which had a relatively high level of expression of endogenous IQGAP3. Two shRNA constructs targeting different IQGAP3 sequences were used, both of which led to efficient downregulation of IQGAP3 in comparison to non-specific controls (Figure 3A). As anticipated, knockdown of IQGAP3 suppressed the proliferation of A549 cells (Figure 3B). Moreover, reduced IQGAP3 expression was also accompanied by decreased migration and invasion in A549 cells (Figure 3C, D). It is well-known that EGFR signaling is critically involved in lung cancer development. We therefore further tested whether IQGAP3 expression can modulate cell sensitivity to EGF stimulus. As shown in Figures 3E and F, knockdown of IQGAP3 caused inhibition of cell migration and invasion similar to that induced by EGF. Taken together, these data suggest IQGAP3 has oncogenic potential.

### Knockdown of IQGAP3 Suppressed Lung Cancer Metastasis

**Figure 4:**
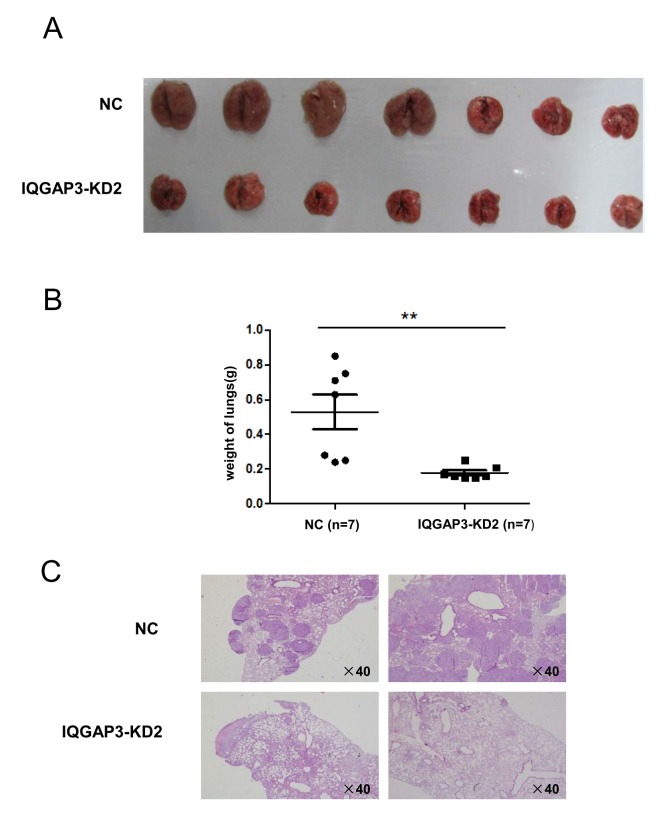
Decreased tumorigenicity of A549 cells *in vivo* with reduced IQGAP3 expression. NOD/SCID mice received an intravenous injection of 1.5×10^6^ A549 cells stably transfected with shIQGAP3 (IQGAP3-KD2) or control vectors (NC), and were sacrificed at day 42 after tumor inoculation (n = 7 for each group). (A) Gross appearance of lung tissues. (B) Lung weight. The horizontal and error bars show the average weight and standard deviation. (C) H&E staining of lung tissues. Four representative fields (2 for each group) are shown. Magnification, ×40. **, *P*<0.01.

In view of the potent impact of IQGAP3 expression on proliferation and migration of cancer cells cultured *in vitro*, we next sought to determine whether it also affected tumorigenesis *in vivo* using an established model of metastatic lung cancer [38], [39]. A549 cells harboring a lentiviral vector expressing IQGAP3-specific shRNA were injected into the tail vein of NOD/SCID mice. Animals were sacrificed at day 42 and the lungs were removed and analyzed. Compared to the mock controls, IQGAP3 knockdown cells appeared to be less tumorigenic *in vivo*, as suggested by the greatly reduced bulk and weight of the affected lungs (Figure 4A, B). Immunohistochemical staining revealed that relatively normal lung structure was maintained in the IQGAP3 knockdown groups, while it was almost completely destroyed by the multiple tumor nodules in the mock control (Figure 4C). Taking the related *in vitro* data into consideration, the reduced tumorigenic potential of IQGAP3 knockdown cells *in vivo* may result from either impaired colonization or proliferation or both.

### IQGAP3 Interacts with ERK1

**Figure 5:**
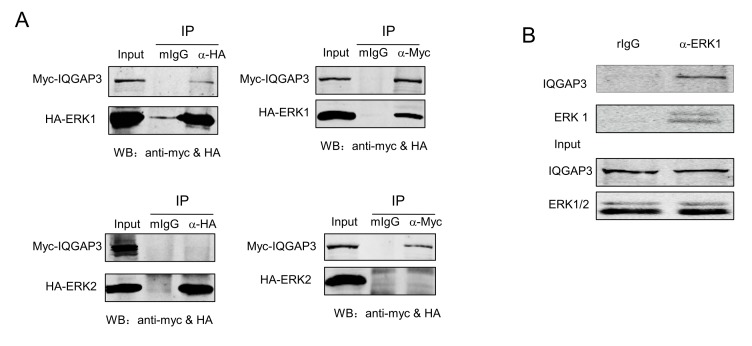
Interaction of IQGAP3 with ERK1. (A) Myc-IQGAP3 and HA-ERK1 or HA-ERK2 constructs were transfected into HEK293T cells. Cell lysate was prepared and immunoprecipitated with mouse anti-HA mAb, anti-Myc mAb or control antibodies (mIgG). The resultant precipitate, together with the unprocessed lysate (Input) was resolved on SDS-PAGE, transferred onto blots and probed with anti-Myc and anti-HA. (B) Interaction of IQGAP3 with ERK1 was also examined in A549 cells with endogenously expressed proteins. Cell lysate was prepared from A549 cells and immunoprecipitated with rabbit anti-ERK1 mAb. The presence of IQGAP3 in the precipitate was evaluated anti-IQGAP3 antibodies. All experiments were repeated at least three times and results from one representative experiment are shown.

As predicted by Scansite (http://scansite.mit.edu/), IQGAP3 contains multiple ERK D domains, which are known to mediate interaction with ERK family members. To explore this possibility, we transfected HEK293T cells with Myc-IQGAP3 and HA-ERK1 or HA-ERK2 constructs. Cell lysate was prepared and immunoprecipitated with anti-HA or anti-Myc antibodies. The resultant precipitate was reciprocally detected with anti-Myc or anti-HA. It was of particular interest that immunoprecipitation of IQGAP3 was observed with ERK1, but not with ERK2 (Figure 5A). Furthermore, we sought to determine whether a similar interaction occurs between endogenously expressed proteins. Cell lysate from A549 cells was immunoprecipitated with rabbit anti-ERK1 mAb and IQGAP3 was indeed detected in the precipitate with anti-IQGAP3 antibody (Figure 5B). These results indicate that IQGAP3 can interact with ERK1, either directly or indirectly through a larger complex.

### IQGAP3 Promotes EGF Induced ERK Phosphorylation

**Figure 6:**
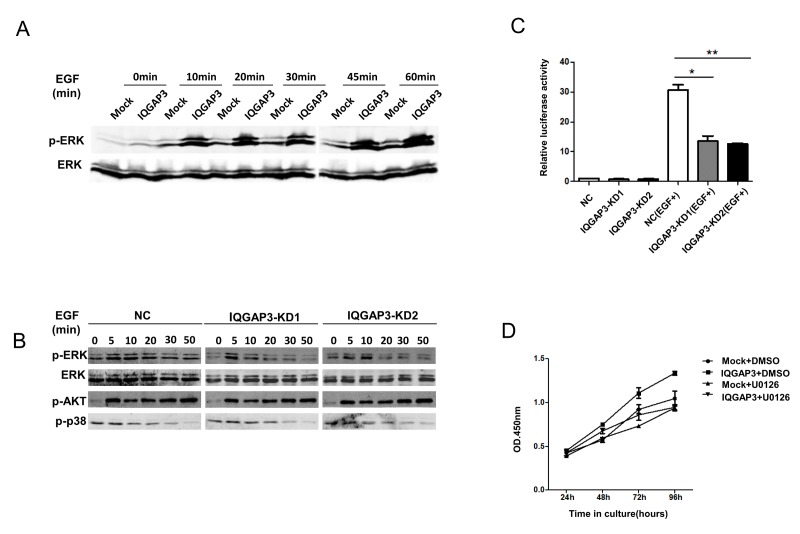
Modulation of EGFR-ERK signaling by IQGAP3. (A) Hela cells transiently transfected with Myc-IQGAP3 or control vector (mock) were serum deprived (0.5% fetal bovine serum) for 24 h before being stimulated with 100 ng/ml EGF. Cells were harvested at different time points and examined for ERK phosphorylation. (B) A549 cells were infected with shIQGAP3 lentivirus (IQGAP3-KD1 or KD2) or control virus (NC) and serum deprived (0.5% fetal bovine serum) prior to stimulation with 100 ng/ml EGF. Levels of phosphorylated ERK, total ERK, phosphorylated p38 and phosphorylated AKT were evaluated with western blot at different time points after EGF stimulation. (C) Luciferase reporter assays were performed to measure Elk1 activity downstream of the cascade of EGFR-ERK signaling in A549 cells transfected with siRNA oligos. (D) Proliferation of Hela cells harboring IQGAP3-expressing or control vectors was assayed using the Cell Counting Kit-8 with addition of 20 µM U0126 or DMSO. Each assay was repeated at least 3 times. The data from one representative experiment are presented as mean±SD. *, *P*<0.05; **, *P*<0.01.

The interaction between IQGAP3 and ERK1 prompted further investigation into the influence of IQGAP3 on ERK activation and signaling. Overexpression of IQGAP3 in Hela cells had minimal effect on the phosphorylation of ERK, in contrast to what has been reported for IQGAP1 [40]. When stimulated with EGF, however, the IQGAP3 transfectants demonstrated greatly enhanced ERK phosphorylation as compared to the mock control (Figure 6A). AKT and p38 activation on the other hand was not altered (data not shown). Consistent with these findings, knockdown of IQGAP3 expression in A549 cells resulted in the attenuation of EGF-induced ERK phosphorylation, while AKT and p38 phosphorylation was not affected (Figure 6B). Moreover, dual luciferase reporter assays showed that knockdown of IQGAP3 inhibits the transcriptional activity of Elk1 which is a hallmark of ERK activation in EGF stimulated A549 cells (Figure 6C). To evaluate the potential correlation between enhanced ERK signaling and accelerated proliferation of IQGAP3-transfected cells, U0126 which is a MEK1/2 inhibitor was added to the culture. As shown in Figure 6D, inhibition of ERK activation abolished the proliferation-promoting effect of IQGAP3. These results support the concept that oncogenic activity of IQGAP3 is likely mediated by enhanced ERK signaling.

## Discussion

To date, three members of the IQGAP family have been described [22]–[24]. There is evidence that IQGAP1 primarily functions as an oncogene [27]. IQGAP2 displays anti-tumor activity, despite its structural similarity to IQGAP1 [33]. Here we demonstrated that IQGAP3 is highly expressed in a large proportion of lung cancer samples. While enforced expression of IQGAP3 causes accelerated proliferation and migration/invasion of cancer cells, suppression of its expression leads to a reduction in tumorigenic potential. At the molecular level, IQGAP3 interacts with ERK1 and promotes EGF-induced activation of ERK, which in turn appears to be closely associated with its tumor-promoting activity.

IQGAP3 is located at 1q21.3, which is a hotspot for gene amplification in cancer [24]. DNA amplification at 1q21.3 has for example been reported to be linked with gastroesophageal carcinoma and infiltrating ductal carcinoma of the breast [41], [42]. In addition, gains at 1q21.3 have a significant correlation with reduced overall survival time in leiomyosarcoma of uterus [43]. Bioinformatics analysis shows that IQGAP3 is upregulated in multiple types of cancers, including ovary, lung, large intestine, gastric, bone marrow and breast malignancies (Figure 1A). The current study specifically addressed its role in lung cancer. Among 25 pairs of lung cancer and adjacent non-cancerous tissues, 20 cancer tissues showed an increase in IQGAP3 mRNA. Upregulation of IQGAP3 expression was confirmed at the protein level by display of stronger immunohistochemical staining in cancer than in non-cancerous tissues in 80 out of 89 lung cancer samples. We speculate that IQGAP3 may represent an important gene which is critically involved in malignancies characterized by 1q21.3 amplification. It would be interesting to determine whether 1q21.3 amplification contributes to the upregulation of IQGAP3 which is found in lung cancer, and whether 1q21.3 amplification observed in gastroesophageal carcinomas or breast cancer is associated with increased IQGAP3 expression. Although in our study, we demonstrate that upreguation of IQGAP3 is a common event in lung cancer, we should mention that there are a few lung cancer samples which show unchanged or even decreased expression of IQGAP3. This is not surprising. For example, Her2, the representing oncogene in breast cancer, is only upregulated in about 20% breast cancer [44], [45]. Some genes, such as p16, which can be upregulated or downregulated in tumors and act as either a tumor suppressor or an oncogene depending on the context of the body [46]. Therefore, whether the different expression pattern of IQGAP3 suggests a more confound regulation and function of IQGAP3 in tumor development needs further studies.

The dysregulation of expression of IQGAP3 in cancer tissue suggests a potential role for this molecule in tumorigenesis. Consistent with this concept, overexpression of IQGAP3 enhanced tumor cell growth, migration and invasion, whereas knockdown of IQGAP3 expression displayed opposite effects. IQGAP3 is therefore functionally similar to IQGAP1, which is known to have oncogenic potential [31], [32]. Like other members of the IQGAP family, IQGAP3 possesses structural motifs which may bind ERK. By reciprocal coimmunoprecipitation, we confirmed the interaction between IQGAP3 and ERK1. The functional relevance of this interaction is supported by the augmented phosphorylation of ERK and increased Elk1 transcriptional activity upon EGF stimulation in IQGAP3-transfected cells. More importantly, the proliferation-promoting effect was almost completely abolished by the ERK signaling inhibitor U0126, indicating that IQGAP3 exerts its function mainly by regulation of ERK signaling. It is interesting to note that despite their functional similarity, IQGAP3 and IQGAP1 display distinctly different specificities for binding partners. While IQGAP1 primarily binds to ERK2, IQGAP3 interacts exclusively with ERK1 [40]. The significance of such selectivity remains to be determined, however we speculate that IQGAP1 and IQGAP3 may cooperate in regulation of ERK signaling, thereby exerting a synergistic effect on tumor progression.

Intravenous inoculation of nude mice has been widely used to assess metastatic potential of cancer cells [38], [39]. Using this model, knockdown of IQGAP3 in lung cancer cells was found to greatly impair its capacity to generate metastatic lesions in the lung. This result is consistent with our *in vitro* data which shows altered cell migration and invasion following manipulation of IQGAP3 expression. However, the specific details of these mechanisms remain unclear. Confocal microscopy revealed that IQGAP3 is enriched at the leading edge of migrating cells (Figure S1), suggesting its possible involvement in formation of leading edges. In addition, gene expression profiling showed an increased level of E-cadherin in IQGAP3 knockdown cells (Figure S2). It is well recognized that E-cadherin is very important for cell adhesion. Downregulation of it along with its associated catenin complex is concomitant with reduced intercellular adhesion and enhanced invasive capacity [47], [48]. As such, IQGAP3 may facilitate cell migration and invasion partly through suppression of E-cadherin expression.

In summary, our studies reveal for the first time the involvement of IQGAP3 in lung cancer development and the potential molecular mechanisms underlying its tumor-promoting activity. These findings expand our knowledge of the complicated role of the IQGAP family in tumorigenesis. Further inquiry into the potential of these molecules as targets for therapeutic invention is warranted.

## Supporting Information

Figure S1 **IQGAP3 was enriched at the leading edge of migrating cells.** A549 cells were placed on cover glass coated with 10 µg/ml fibronectin (Sigma–Aldrich). In order to leave a 6 mm-wide wet section with adhering cells, when the cells had adhered, the ends of the cover glass were wiped dry. After the cover glass was inverted and placed in the Zigmond chamber, serum-free RPMI 1640 containing 30ng/ml EGF was added to one side of the chamber and serum-free RPMI 1640 was added to the other side. The chamber was then incubated for 1 h at 37°C. Cells were fixed with 4% paraformaldehyde for 15 min at 37°C and were permeabilized for 5 min with 0.1% Triton X-100 (Sigma–Aldrich). Samples were blocked for 1 h with PBS containing 5% bovine serum albumin and probed with the anti-IQGAP3 antibody at 4°C overnight. After thorough washing, samples were incubated with secondary antibody and phalloidin (Sigma–Aldrich) at room temperature for 1 h. Nuclear DNA was labeled with Hoechst 33342 (Life Technologies). Cells were imaged with a Leica TCS SP5 confocal microscope (Leica Microsystems). Scale bar, 10 µm.
(TIF)

Figure S2 **Inhibition of IQGAP3 expression was accompanied by upregulation of E-cadherin.** A549 cells were infected with control (NC) or two different shIQGAP3 lentiviruses. Cell lysate was probed for IQGAP3, E-cadherin and GAPDH expression by Western blotting.
(TIF)

Table S1 **The correlation of IQGAP3 protein expression and clinico-pathologic characteristics in patients with lung cancer.**
(DOCX)
