# Supplementary figures and images for: IQGAP3 Promotes EGFR-ERK Signaling and the Growth and Metastasis of Lung Cancer Cells
Source: PLoS One. 2014 May 21;9(5):e97578. doi: 10.1371/journal.pone.0097578 (PMC4029748; doi:10.1371/journal.pone.0097578)

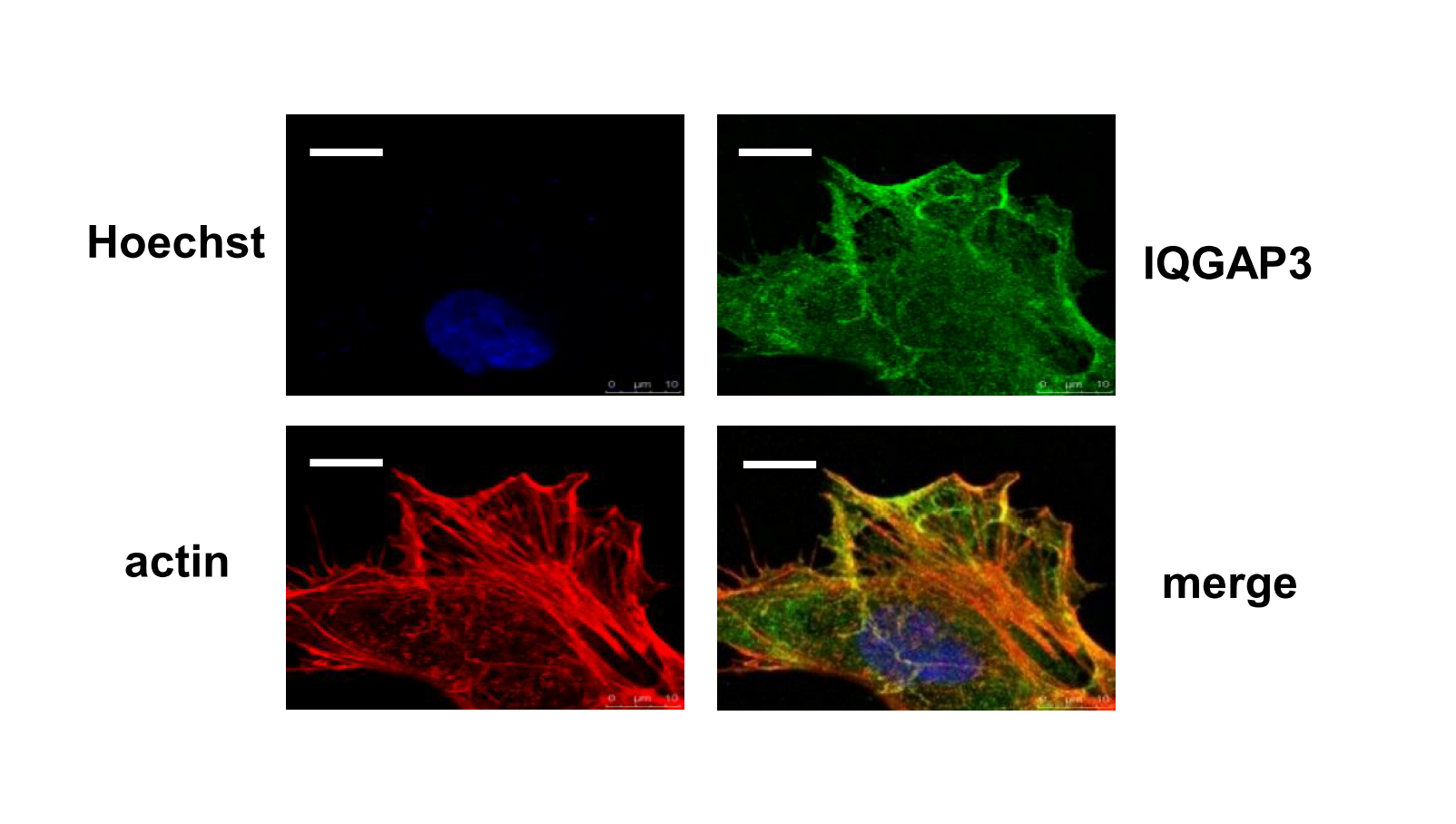

Supplement: Figure S1 — IQGAP3 was enriched at the leading edge of migrating cells. A549 cells were placed on cover glass coated with 10 µg/ml fibronectin (Sigma–Aldrich). In order to leave a 6 mm-wide wet section with adhering cells, when the cells had adhered, the ends of the cover glass were wiped dry. After the cover glass was inverted and placed in the Zigmond chamber, serum-free RPMI 1640 containing 30ng/ml EGF was added to one side of the chamber and serum-free RPMI 1640 was added to the other side. The chamber was then incubated for 1 h at 37°C. Cells were fixed with 4% paraformaldehyde for 15 min at 37°C and were permeabilized for 5 min with 0.1% Triton X-100 (Sigma–Aldrich). Samples were blocked for 1 h with PBS containing 5% bovine serum albumin and probed with the anti-IQGAP3 antibody at 4°C overnight. After thorough washing, samples were incubated with secondary antibody and phalloidin (Sigma–Aldrich) at room temperature for 1 h. Nuclear DNA was labeled with Hoechst 33342 (Life Technologies). Cells were imaged with a Leica TCS SP5 confocal microscope (Leica Microsystems). Scale bar, 10 µm. (TIF) [file pone.0097578.s001.tif]

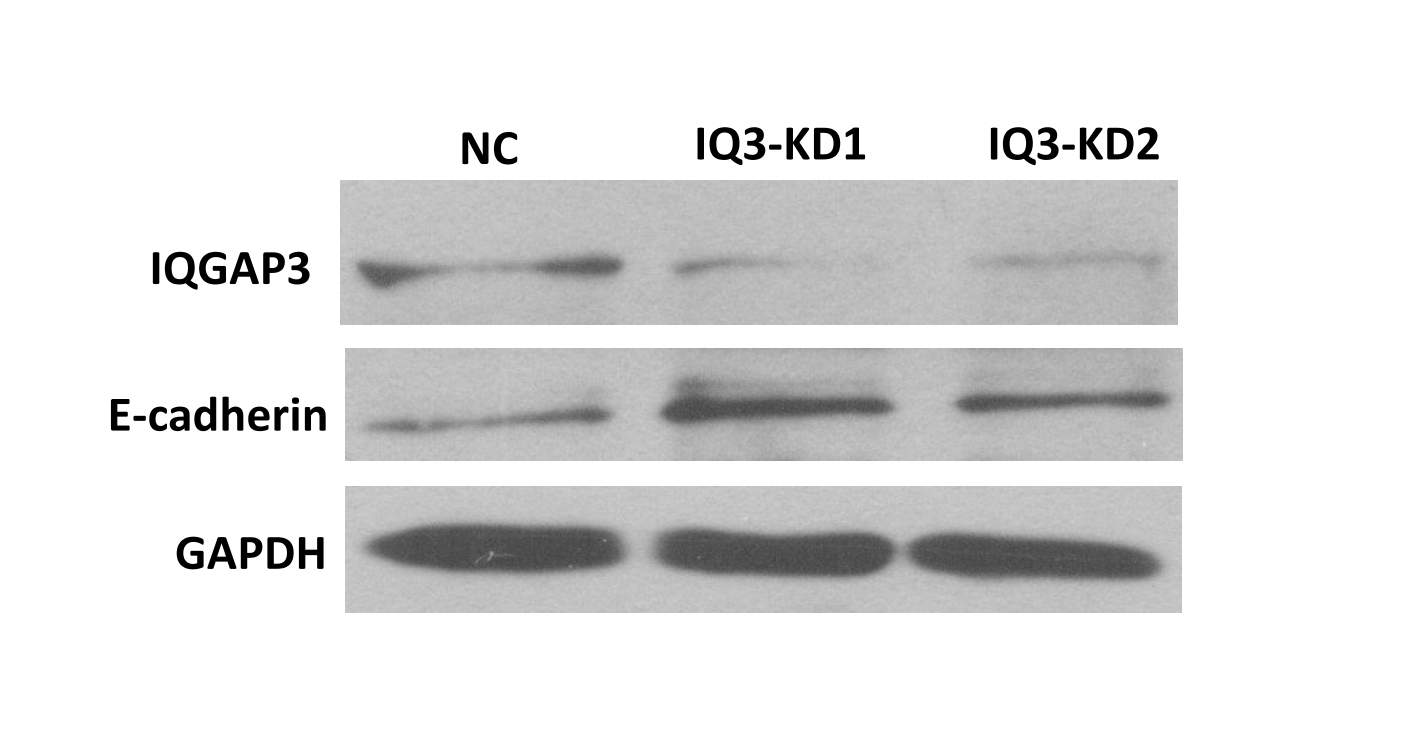

Supplement: Figure S2 — Inhibition of IQGAP3 expression was accompanied by upregulation of E-cadherin. A549 cells were infected with control (NC) or two different shIQGAP3 lentiviruses. Cell lysate was probed for IQGAP3, E-cadherin and GAPDH expression by Western blotting. (TIF) [file pone.0097578.s002.tif]
