# Supplementary material for: IQGAP3 Promotes EGFR-ERK Signaling and the Growth and Metastasis of Lung Cancer Cells
Source: PLoS One. 2014 May 21;9(5):e97578. doi: 10.1371/journal.pone.0097578 (PMC4029748; doi:10.1371/journal.pone.0097578)
Supplement: Table S1 — The correlation of IQGAP3 protein expression and clinico-pathologic characteristics in patients with lung cancer. (DOCX) [file pone.0097578.s003.docx]

Table S1 The correlation of IQGAP3 protein expression and clinico-pathologic characteristics in patients with lung cancer

|  | Categorization |  | IQGAP3 | | |  |
| --- | --- | --- | --- | --- | --- | --- |
|  |  | n | Adj>LC | Adj=LC | Adj<LC | *P*-value |
|  |  |  |  |  |  |  |
| Total |  | 89 | 2(2%) | 7(8%) | 80(90%) |  |
| Age | <60 | 32 | 0 | 3(9%) | 29(91%) | 0.705 |
|  | ≧60 | 56 | 1(2%) | 4(7%) | 51(91%) |  |
|  | Unknown | 1 | 1(100%) | 0 | 0 |  |
| Gender | Male | 52 | 1(2%) | 7(13%) | 44(85%) | 0.052 |
|  | Female | 35 | 0 | 0 | 35(100%) |  |
|  | Unknown | 2 | 1(50%) | 0 | 1(50%) |  |
| Histology | adenocarcinoma | 46 | 1(2%) | 2(4%) | 43(94%) | 0.681 |
|  | squamous cell carcinoma | 38 | 1(3%) | 4(10%) | 33(87%) |  |
|  | others | 5 | 0 | 1(20%) | 4(80%) |  |
| Pathological grading | Ⅰ | 25 | 0 | 3(12%) | 22(88%) | 0.549 |
|  | Ⅱ | 52 | 2(4%) | 3(6%) | 47(90%) |  |
|  | Ⅲ | 9 | 0 | 0 | 9(100%) |  |
|  | Unknown | 3 | 0 | 1(33%) | 2(67%) |  |
| Metastasis | yes | 23 | 1(4%) | 1(4%) | 21(92%) | 0.576 |
|  | no | 66 | 1(2%) | 6(9%) | 59(89%) |  |
